# Submandibular Lateral Ectopic Thyroid Tissue: Ultrasonography, Computed Tomography, and Scintigraphic Findings

**DOI:** 10.1155/2015/769604

**Published:** 2015-11-08

**Authors:** Metin Çeliker, Fatma Beyazal Çeliker, Arzu Turan, Mehmet Beyazal, Hatice Beyazal Polat

**Affiliations:** ^1^Department of Ear, Nose and Throat, Faculty of Medicine, Recep Tayyip Erdoğan University, 53020 Rize, Turkey; ^2^Department of Radiology, Faculty of Medicine, Recep Tayyip Erdoğan University, 53020 Rize, Turkey; ^3^Department of Radiology, Muğla Yatağan State Hospital, 48500 Muğla, Turkey; ^4^Department of Internal Medicine, Faculty of Medicine, Recep Tayyip Erdoğan University, 53020 Rize, Turkey

## Abstract

Ectopic thyroid can be encountered anywhere between the base of tongue and pretracheal region. The most common form is euthyroid neck mass. Herein, we aimed to present the findings of a female case with ectopic thyroid tissue localized in the left submandibular region. A 44-year-old female patient, who underwent bilateral subtotal thyroidectomy four years ago with the diagnosis of multinodular goiter, was admitted to our hospital due to a mass localized in the left submandibular area that gradually increased in the last six months. Neck ultrasonography, contrast-enhanced computed tomography, and scintigraphic examination were performed on the patient. On thyroid scintigraphy with Tc-99m pertechnetate, thyroid tissue activity uptake showing massive radioactivity was observed in the normal localization of the thyroid gland and in the submandibular localization. The focus in the submandibular region was excised. Pathological examination of the specimen showed normal thyroid follicle cells with no signs of malignancy. The submandibular mass is a rarely encountered lateral ectopic thyroid tissue. Accordingly, ectopic thyroid tissue should also be considered in the differential diagnosis of masses in the submandibular region.

## 1. Introduction

Ectopic thyroid tissue is derived from incomplete migration of thyroid gland and can be found anywhere between the base of tongue and pretracheal region. Ectopic thyroid tissue most commonly appears in the midline in the cervical region (90% of the cases) [1]. Its prevalence is approximately 1/100,000–1/300,000 [2]. Lateral ectopic thyroid tissue is a much less common condition [3]. Herein, we aimed to present the findings of a female case with ectopic thyroid tissue localized in the left submandibular region.

## 2. Case Presentation

**Figure 1:**
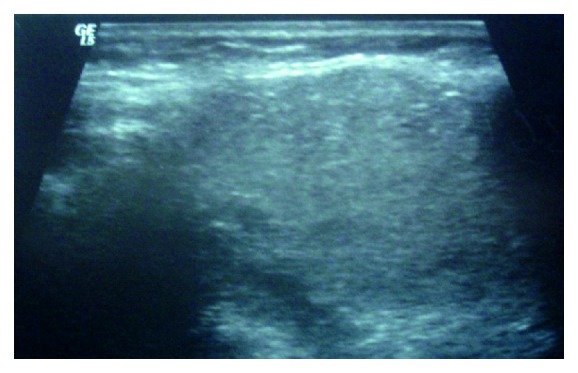
Ultrasonography findings; a 30 × 25 mm mass lesion with parenchymal echo pattern containing cystic degenerative areas in the left submandibular region.

**Figure 2:**
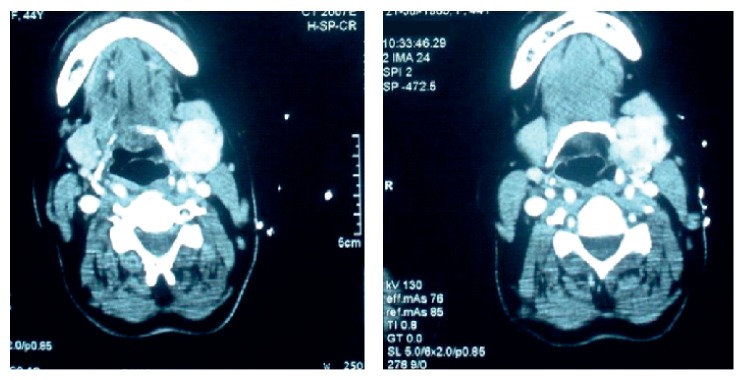
Postcontrast computed tomography findings; a well-circumscribed left submandibular mass.

**Figure 3:**
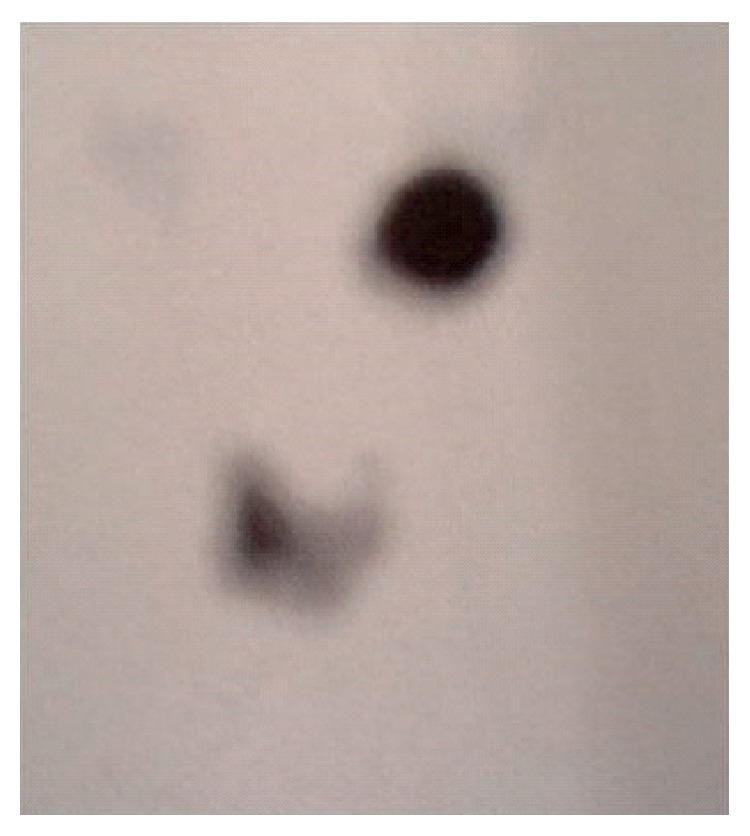
Radioactivity uptake in the normal localization of the thyroid gland and in the submandibular localization on thyroid scintigraphy with Tc-99m pertechnetate.

A 44-year-old female patient, who underwent bilateral subtotal thyroidectomy four years ago with the diagnosis of multinodular goiter, was admitted to our hospital due to a mass localized in the left submandibular area that gradually increased in the last six months. Informed consent was obtained from the patient. Neck ultrasonography (US), contrast-enhanced computed tomography (CT), and scintigraphic examination were performed on the patient. US examination revealed newly developed nodules with heterogeneous echo pattern in residual thyroid tissue in the normal localization, as well as a 30 × 25 mm mass lesion with parenchymal echo pattern containing cystic degenerative areas in the left submandibular region (Figure 1). On the neck CT, there was residual thyroid tissue with heterogeneous density in normal localization and a well-circumscribed left submandibular mass with equal density (Figure 2). On thyroid scintigraphy with Tc-99m pertechnetate, thyroid tissue activity uptake showing massive radioactivity was observed in the normal localization of the thyroid gland and in the submandibular localization (Figure 3). Preoperatively, thyroid hormone levels and biochemical values of the patient were normal. The focus in the submandibular region was excised. The patient had spontaneous euthyroidism in the postoperative period. Pathological examination of the specimen showed normal thyroid follicle cells with no signs of malignancy. Thus, it was confirmed that the submandibular mass was a rarely encountered lateral ectopic thyroid tissue.

## 3. Discussion

Abnormalities of thyroid gland during embryologic development and migration may result in ectopic thyroid gland. Ectopic thyroid gland can be seen in any localization from the base of the tongue to the mediastinum in the midline on the neck [4, 5]. Normally, migration of the thyroid gland is from the foramen cecum to the pretracheal position [6]. In addition to normal migration pathway of the thyroid gland, ectopic thyroid tissue can be seen even in mediastinal, intracardiac, gastrointestinal, and intraperitoneal localizations [2, 4, 5]. Ectopic thyroid tissue is mostly (90%) localized in sublingual position. Ectopic thyroid tissue in the submandibular space with the thyroid gland in its normal location is an extremely rare phenomenon [4]. There are few available case reports in the literature on this issue [2–9].

Ectopic thyroid gland is more prevalent in females than in males and is usually asymptomatic [10]. Asymptomatic ectopic thyroid tissue may become symptomatic particularly in the adolescence and pregnancy period due to increase in thyroid stimulating hormone level and to thyroid tissue hyperplasia [11]. Ectopic thyroid tissue is usually hypoactive but may rarely be hyperactive. Etiology of ectopic thyroid tissue is unclear; however, it has been suggested that gene mutations play a role [12]. “Thyroid transcription factor 2” (TTF-2) mutation is associated with thyroid agenesis and other defects. “*PAX8* gene” mutation has been found to be associated with various forms of thyroid dysgenesis, whereas* TTF-1* gene mutation has been found to be associated with thyroid agenesis or dysgenesis. These gene mutations also cause ectopic migration [12]. In 70% of the ectopic thyroid tissues, only thyroid tissue is present [13]. All thyroid gland diseases can be seen also in ectopic thyroid tissue. All diseases that involve thyroid tissue in its normal localization also involve ectopic thyroid tissue. Thyroglossal duct cyst, hyperplastic lymphoid tissue, lymphangioma, fibroma, lipoma, dermoid cyst, squamous cell carcinoma, minor salivary gland tumor, lymphoma, and vascular tumors should be considered in the differential diagnosis [7, 14]. Metastatic thyroid carcinoma should also be taken into account in the differential diagnosis and it should be considered first among the diseases that will be excluded in the differential diagnosis.

Ultrasonography, scintigraphy, CT, and magnetic resonance imaging (MRI) are the methods that can be used in the diagnosis. Thyroid scintigraphy is a sensitive and specific method in determining that thyroid gland is not in its normal localization. US and CT are beneficial in the diagnosis but have low sensitivity and specificity. The role of imaging in the primary differential diagnosis of ectopic tissues is to detect the normal thyroid tissue in its lodge and to decide removal or transplantation of the whole ectopic tissue. US is the first imaging method to be performed; if noncontrast CT is used, ectopic tissues are observed to be denser compared to the neighboring muscle. In CT, the contrast uptake of the ectopic tissue is observed to be homogenous. In MRI, ectopic tissue is observed to be iso- or hyperintense compared to muscles. In addition to imaging of the normal thyroid tissue, thyroid scintigraphy is also important to show the functions of the lingual thyroid tissue. Differential diagnosis of ectopic thyroid tissue from other diseases varies according to the lodge where it is located. While the differential diagnosis of an intrathyroidal ectopic tissue in a child may be thymus diseases, the differential diagnosis of ectopic tissue in the mediastinal lodge may be lymphoma and hematological diseases [15]. Patients are usually asymptomatic. However, dysphagia, dysphonia, dyspnea, heart failure, and bleeding may be encountered depending on the localization of the thyroid gland [5, 11]. Moreover, compression on surrounding tissue and malignant degeneration can also be observed [5, 13]. Less than 1% of ectopic thyroids have malignant transformation, of which papillary carcinoma is the most common (approximately 85%) malignancy [7]. Although it has been reported that possibility of malignant degeneration in ectopic thyroid tissue is not higher than in normal tissues [16], there are recent case reports on malignant degeneration in ectopic thyroid tissues within a branchial cleft cyst [17].

In conclusion, lateral ectopic thyroid tissue is a very rare condition found most commonly in submandibular localization and the number of cases reported in the literature is limited. Less than 1% of ectopic thyroids have malignant transformation, of which papillary carcinoma is the most common (approximately 85%) malignancy [7]. US and CT are the auxiliary methods in the diagnosis. Scintigraphy, the guide in demonstrating functional thyroid tissue and in planning treatment, should certainly be performed.
